# Neutrophil Fate and Function in Gout: From Sterile Inflammation to Resolution

**DOI:** 10.3390/ijms27187979

**Published:** 2026-09-08

**Authors:** Yundong Xu, Qianqian Yang, Jian Zhang, Rong Wang, Sanjin Zeng, Dashuai Tuo, Niqin Xiao, Heguo Yan, Bingbing Chen, Shengyi Zhao, Zhaohu Xie, Xiaoyu Zhang, Zhaofu Li

**Affiliations:** 1 The First Clinical Medical College, Yunnan University of Chinese, Kunming 650500, China; xuxu1204@126.com (Y.X.);; 2 Joint Graduate School of Traditional Chinese Medicine of China, Suzhou 215106, China

**Keywords:** gout, monosodium urate crystals, neutrophils, sterile inflammation, neutrophil extracellular traps, inflammasome, efferocytosis, extracellular vesicles, inflammation resolution

## Abstract

Gout is a prototypical sterile inflammatory disease caused by the deposition of monosodium urate (MSU) crystals in joints and periarticular tissues. Although acute gout flares are characterized by rapid and intense neutrophil-dominated inflammation, they often resolve spontaneously, suggesting that neutrophils may contribute not only to inflammatory amplification but also to endogenous resolution. In this review, we summarize current evidence on the multifaceted roles of neutrophils in gout across different stages of disease. We discuss how MSU crystals activate innate immune signaling and promote neutrophil recruitment, activation, and effector responses, thereby driving acute inflammation. We further examine the diverse neutrophil programs involved in gout, including apoptosis, necroptosis, pyroptosis, autophagy, efferocytosis, and NET release. Particular attention is given to the dual roles of NETs in gout, as they can both amplify inflammation and, in their aggregated form, facilitate cytokine degradation, crystal sequestration, and inflammation resolution. In addition, we address the potential contribution of neutrophil-derived extracellular vesicles and neutrophil-associated mechanisms to tophus formation and chronic disease progression. By integrating recent advances and ongoing controversies, this review highlights neutrophils as dynamic regulators of both inflammatory injury and resolution in gout, and underscores their potential as therapeutic targets in immune-mediated crystal inflammation.

## 1. Introduction

Gout is an inflammatory disease induced by monosodium urate (MSU) crystals, primarily characterized by recurrent acute inflammation of joints and tissues. This condition not only gradually leads to the formation of tophi but can also result in joint damage and deformity [1]. Epidemiological data show that the prevalence of gout has reached 1% to 6.8% worldwide, with an increasing trend year by year [2]. It is noteworthy that neutrophils, macrophages, and T lymphocytes play crucial roles in the onset and resolution of gout [3]. Additionally, some inflammatory cytokines, such as factors from the interleukin (IL)-1 family, are involved in the inflammatory process of gout. However, their specific mechanisms remain to be elucidated [4]. Gout flares cause joint redness, swelling, and severe pain, symptoms that typically resolve spontaneously within 7–10 days. However, without continuous treatment, the frequency and duration of gout flare may increase. More seriously, gout can be associated with several comorbidities, including hypertension, diabetes, and cardiovascular diseases [2].

High serum uric acid levels are a critical factor in the onset of gout [5]. MSU crystals act as endogenous danger signals in this context, activating the body’s innate immune system and subsequently inducing neutrophil chemotaxis and recruitment. During this process, various biological processes of neutrophils, such as apoptosis, necrosis, pyroptosis, autophagy, and efferocytosis, play different roles in inflammation. Under MSU stimulation, neutrophils can release neutrophil extracellular traps (NETs), which are extracellular chromatin structures decorated with granular proteins [6]. NETosis generally refers to the death-associated process of NET release, whereas NET release can also occur through non-lytic pathways. Accordingly, in this review, “NET formation” or “NET release” is used as an umbrella term when the underlying mode of neutrophil death has not been established [6,7,8]. MSU crystals can promote NET release by neutrophils, thereby amplifying the inflammatory response; meanwhile, NETs can aggregate and degrade cytokines and chemokines and encapsulate MSU crystals to limit inflammation. This dual proinflammatory and anti-inflammatory effect of NETs induced by MSU crystals may underlie tophi formation [7,8]. Although neutrophils are classically viewed as proinflammatory cells, accumulating evidence suggests that they may also participate in the spontaneous resolution of gout in a context-dependent manner, particularly through apoptosis/efferocytosis and aggregated NET formation that can sequester MSU crystals and degrade proinflammatory mediators [9,10,11]. This paper primarily explores the specific mechanisms of neutrophils in gout, aiming to provide new insights into the prevention and treatment of gout by analyzing and summarizing relevant research findings.

## 2. Innate Immunity of Neutrophils During Gout Flare

The natural course and clinical manifestations of gout can be broadly divided into four stages: asymptomatic hyperuricemia, acute gouty arthritis, gout remission, and chronic tophaceous gout [1]. Neutrophils play different roles at each stage. During an acute flare, MSU crystal recognition is better understood as a two-step inflammatory process rather than as a single linear CD14-TLR-NLRP3 pathway. First, priming signals, including TLR2/TLR4-dependent MyD88-IKK-NF-κB signaling in some experimental settings, can increase the transcription of NLRP3 and pro-IL-1β [12]. CD14 has been reported to mediate the inflammatory potential of MSU crystals and may facilitate crystal-cell interactions, but it should not be described simply as a molecule that directly links MSU crystals to TLR activation [13]. Second, after phagocytosis, MSU crystals promote lysosomal injury, ion fluxes, mitochondrial stress, and ROS-associated signals, leading to NLRP3 inflammasome assembly, caspase-1 activation, and maturation of IL-1β and IL-18 [14,15].

**Figure 1:**
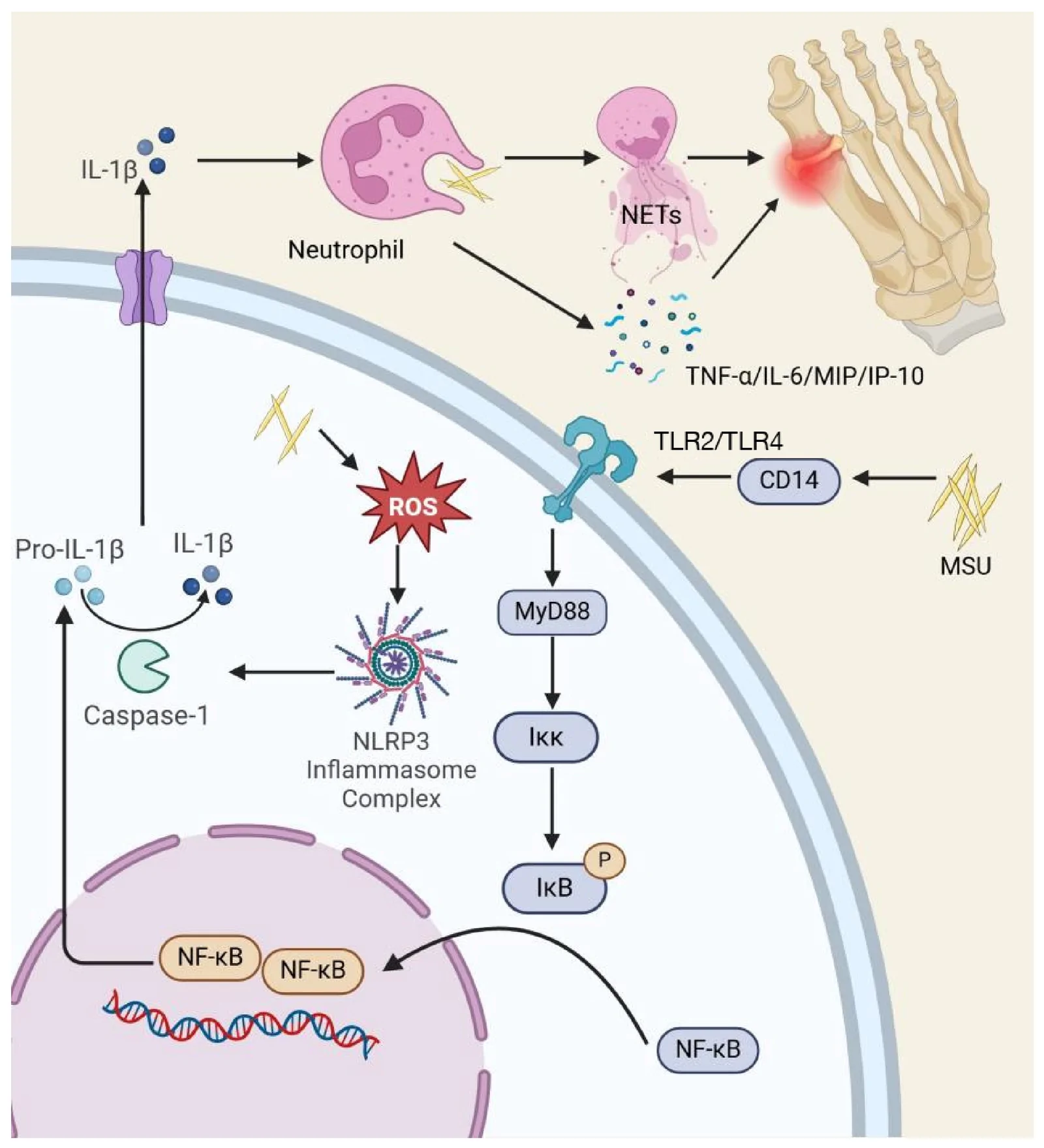
MSU induces inflammation through the TLR2/TLR4/MyD88/NF-κB signaling pathway. Once IL-1β binds to its receptor, it triggers downstream signaling, leading to the recruitment of neutrophils and other immune cells to the site of crystal deposition. During the phagocytosis of MSU crystals, neutrophils release various inflammatory mediators such as tumor necrosis factor (TNF)-α, IL-6, and activators like macrophage inflammatory protein and interferon-γ-inducible protein-10, all of which drive the development of the immune response. IL-1β amplifies local inflammation through IL-1R-MyD88-dependent signaling, promotes chemokine-driven neutrophil recruitment, and may enhance MSU-induced NET formation together with direct MSU-neutrophil interactions, autophagy-related signaling, ROS-dependent pathways, and caspase-11/cofilin signaling. Although NETs play an essential role in defense mechanisms, they can also cause damage to surrounding tissues.

Mature IL-1β then acts mainly through the IL-1 receptor-MyD88 axis to amplify local inflammation and promote neutrophil recruitment, rather than functioning as a classical direct neutrophil chemoattractant [16]. IL-1β-dependent signaling induces inflammatory and chemokine networks that support the migration of neutrophils and monocytes to sites of crystal deposition. In relation to NET formation, macrophage-derived IL-1β has been shown to enhance MSU-triggered NET release, and IL-1β-associated autophagy signaling has been linked to NET formation in gout; however, NET release also depends on direct neutrophil responses to MSU crystals and additional pathways such as ROS, autophagy, and caspase-11/cofilin signaling [17,18,19]. During MSU crystal phagocytosis, activated neutrophils release inflammatory mediators, including TNF-α, IL-6, chemokines, ROS, and granule proteins, which further amplify local inflammation and tissue injury. The relationships among MSU crystal recognition, inflammatory priming and activation, IL-1β-dependent neutrophil recruitment, and NET release are summarized in Figure 1.

The activation of neutrophils induced by MSU crystals is a critical step in the inflammatory cascade of gout. Neutrophils are the most abundant leukocytes in human circulation and one of the dominant immune cell populations in synovial fluid during acute gout flares [20,21,22]. Their recruitment from the circulation into MSU-inflamed joints is a multistep process involving endothelial adhesion. Endothelial P-selectin and E-selectin mediate the initial capture and rolling of neutrophils, whereas chemokine-induced activation of β2 integrins, including LFA-1 and Mac-1, promotes firm adhesion through binding to endothelial ICAM-1 and facilitates subsequent transendothelial migration [23]. These cells form the first line of defense in cell-mediated immunity, responding rapidly to microbial invasion. These cells can perform antimicrobial functions through phagocytosis, killing, and degradation of pathogens [23].

Additionally, neutrophils can precisely regulate their granule enzymes, release immunoregulatory cytokines and chemokines, and interact with other immune system components, further recruiting inflammatory cells. However, neutrophils’ uncontrolled release of inflammatory factors can cause significant tissue damage at the inflammation site [24].

Gout flare is closely related to various inflammatory cytokines [25]. MSU crystals can activate the NLRP3 inflammasome and further trigger caspase-1 activation and the maturation of IL-1β and IL-18, thereby promoting the massive influx of neutrophils and monocytes into the affected joints [26]. The various inflammatory factors secreted by neutrophils play different roles in gout flare [27]. Among these, IL-1β and IL-8 promote the chemotaxis of macrophages and neutrophils, while TNF-α promotes neutrophil survival and the synthesis of inflammatory factors, and IL-17 upregulates the expression of inflammatory factors and promotes neutrophil chemotaxis [28,29]. In contrast, IL-10 and transforming growth factor (TGF)-β inhibit the production and release of inflammatory factors [30,31]. Neutrophils are key cell populations in maintaining and amplifying gout flare. Activated neutrophils release proinflammatory cytokines, anti-inflammatory cytokines, and various CXC and CC chemokines and TNF family members [32]. Studies have also found that neutrophil activation markers, peroxidase, and calprotectin are associated with the characteristics of active polyarticular gout [21].

## 3. Neutrophil Death Mechanisms and Gout

### 3.1. Neutrophil Apoptosis, Phagocytosis, and Efferocytosis

Neutrophil apoptosis followed by macrophage clearance marks the transition from the active phase of disease to the resolution of inflammation. Apoptosis is a programmed cell death mechanism [33] and is considered the least damaging to surrounding tissues among all neutrophil death modalities [34]. Apoptosis leads to structural changes in the cell, such as cell shrinkage and internal structural alterations, and ultimately results in clearance by other cells [35]. Apoptosis occurs via two pathways: an intrinsic pathway triggered by intracellular stimuli such as deoxyribonucleic acid (DNA) damage and an extrinsic pathway initiated by extracellular death receptors; both pathways depend on the activation of caspases [36]. Foxo3a is a forkhead box O family transcription factor that prevents Fas ligand-induced neutrophil apoptosis in inflammatory arthritis, thereby prolonging neutrophil survival and contributing to persistent inflammation [37]. In mouse models of gout, inhibition of phosphoinositide 3-kinase (PI3K) can promote inflammation resolution by inducing neutrophil apoptosis through inhibition of PI3K-γ or PI3K-δ isoforms, reducing the production of proinflammatory mediators [38].

During the resolution of inflammation, apoptotic neutrophils are major targets for macrophage-mediated efferocytosis [39]. Efferocytosis, clearing apoptotic cells by phagocytic cells, is crucial in resolving inflammation and promoting tissue repair during the proliferation and remodeling stages [40]. This process activates the synthesis of lipoxins, DHA protectins, and E-series resolvins while reducing the production of classical proinflammatory eicosanoids [40,41]. These pro-resolving lipid mediators collectively decrease vascular permeability, inhibit further neutrophil migration, recruit non-inflammatory monocytes, induce neutrophil apoptosis, and promote their efferocytosis, creating a positive feedback loop that favors resolution. In addition to being cleared by macrophages, neutrophils can also phagocytose apoptotic cells under inflammatory conditions, although this function is less well characterized than macrophage-mediated efferocytosis. The apoptotic targets described in experimental studies mainly include apoptotic neutrophils and other apoptotic inflammatory cells or cell fragments present in the local inflammatory exudate. This neutrophil-mediated uptake can be enhanced by inflammatory stimuli such as GM-CSF, TNF-α, IFN-γ, and TLR agonists [42,43], and may reduce selected proinflammatory neutrophil functions after apoptotic cell uptake [42,43]. In MSU crystal-induced inflammation, the engulfment of apoptotic neutrophils by neutrophils, also described as neutrophil “cannibalism”, has been linked to TGF-β1 production and self-limitation of neutrophil inflammatory activity [44]. In response to tissue injury, local neutrophils initiate a highly coordinated form of chemotaxis involving sequential signals from chemokines, lipids, and other chemotactic agents via autocrine and paracrine mechanisms [45]. This phenomenon, known as neutrophil “swarming”, results in a substantial accumulation of neutrophils at the injury site, often surpassing the number of macrophages present. Although research on efferocytosis in gout is still limited, studies in other types of arthritis have provided substantial insights. For instance, in rheumatoid arthritis, impaired efferocytosis has been linked to persistent inflammation and tissue damage [46]. This existing body of research offers a valuable framework for exploring the role of efferocytosis in gout.

TGF-β1 is considered an important anti-inflammatory mediator during the resolution phase of gout, but its source should not be attributed exclusively to neutrophils. Engulfment of apoptotic cells by macrophages can induce anti-inflammatory mediator production, including TGF-β and TGF-β1, and promote inflammation resolution [47,48]. In gout, TGF-β1 levels increase in synovial fluid during the resolution phase of acute attacks [49]. Macrophages have also been shown to release TGF-β1 during the resolution of MSU crystal-induced inflammation, indicating that macrophages are an important non-neutrophil source of this mediator [50]. Meanwhile, neutrophils may contribute to local TGF-β1 production both indirectly, by undergoing apoptosis and becoming targets for efferocytosis, and directly, in settings where they engulf apoptotic neutrophils during MSU-induced inflammation [44]. Consistent with the anti-inflammatory role of this pathway, exogenous TGF-β1 can reduce inflammatory cell influx in experimental MSU crystal-induced inflammation [51]. Therefore, TGF-β1-mediated resolution in gout should be viewed as a multicellular process involving macrophage efferocytosis, apoptotic neutrophil clearance, and neutrophil cannibalism, rather than as a pathway driven solely by neutrophil-derived TGF-β1.

### 3.2. Neutrophil Necroptosis and Gout

**Figure 2:**
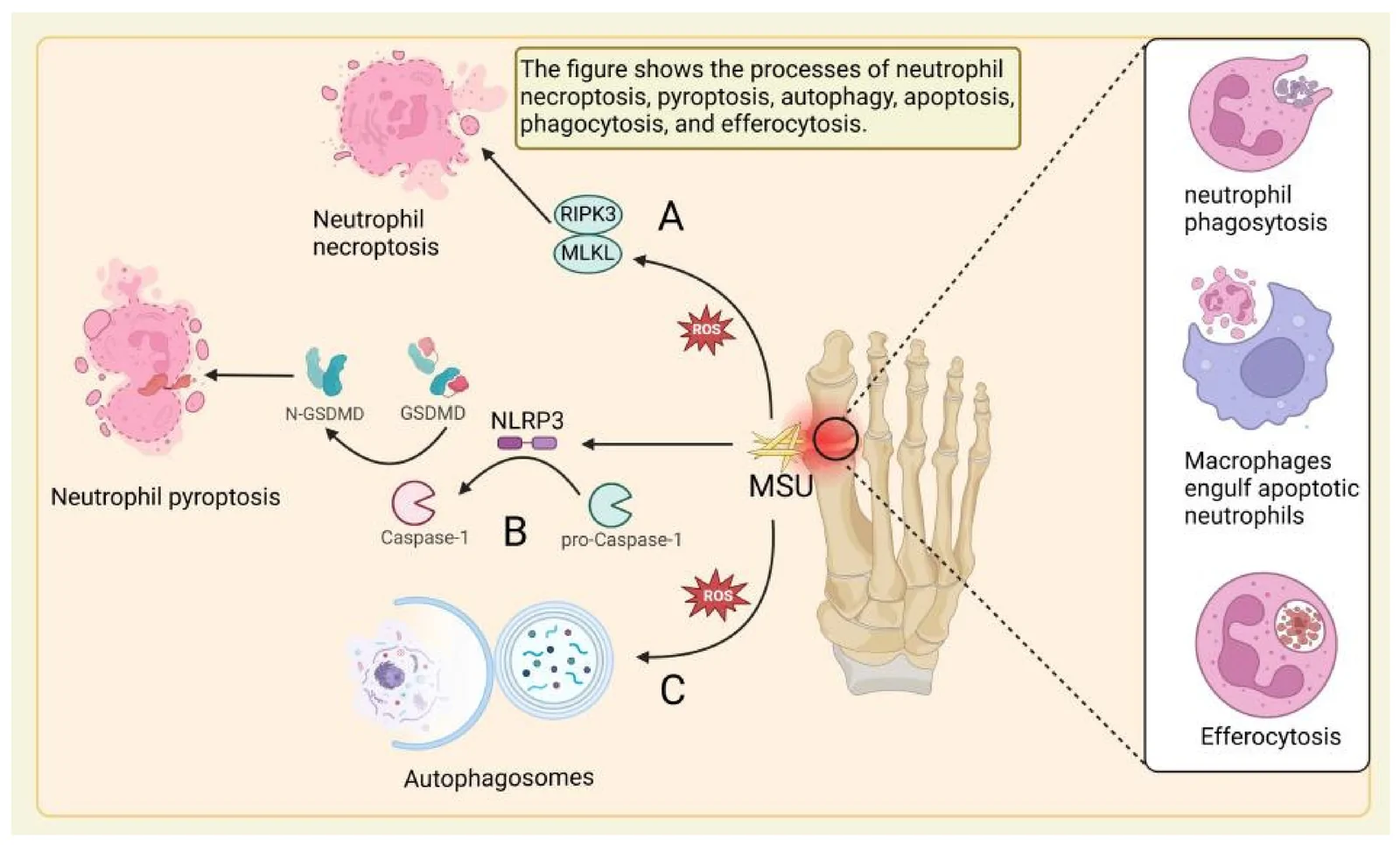
Neutrophils intervene in gout inflammation from multiple angles and must be promptly cleared after participating in the inflammatory response: (**A**) MSU can activate the RIPK3-MLKL complex through ROS, leading to neutrophil necroptosis. (**B**) In the canonical pyroptosis pathway, inflammasome assembly activates caspase-1, which cleaves GSDMD. The N-terminal fragment of GSDMD then oligomerizes in the plasma membrane to form pores, leading to cell swelling, membrane rupture, and release of intracellular contents. (**C**) When neutrophils are stimulated by MSU and ROS, autophagy occurs. The ingested MSU crystals damage the lysosomal membrane, leading to the abnormal release of lysosomal contents.

Necroptosis is another type of programmed cell death [52], where receptor-interacting protein kinase 3 (RIPK3) and mixed lineage kinase domain-like protein (MLKL) play crucial roles [53]. It has been demonstrated that MSU crystals can induce necroptosis via the RIPK3-MLKL pathway [50]. Mechanistically, RIPK3 phosphorylates MLKL, and phosphorylated MLKL oligomerizes and translocates to the plasma membrane, where it disrupts membrane integrity and ion homeostasis, leading to cell swelling, plasma-membrane rupture, and release of intracellular contents [54,55]. In neutrophils, this lytic death program is accompanied by ROS/NADPH oxidase activation and can promote NET-like chromatin release, thereby amplifying local inflammation rather than silent cell clearance [52,54,56]. In the presence of MSU crystals, the activation of necroptosis is closely associated with the generation of intracellular reactive oxygen species (ROS). Although the exact mechanism by which ROS activates the RIPK3-MLKL complex is not fully understood, the generation of ROS within cells is critical for initiating necroptosis (Figure 2A). Notably, ROS appears to be required for efficient neutrophil necroptosis, which is consistent with reduced necroptotic responses in ROS-deficient settings [52,54].

In the context of MSU presence, there is a connection between necroptosis and the release of NETs. This process, accompanied by ROS production, triggers the activation of the NADPH oxidase complex. Interestingly, inhibition of RIPK3 or other necroptosis inhibitors can reduce MSU-induced NET formation, partially confirming the link between necroptosis and NETosis [55]. Neutrophils undergoing necroptosis can also activate NF-κB in adjacent macrophages or fibroblasts, thereby promoting the production of inflammatory factors such as IL-6, IL-8, and TNF-α. These immunomodulatory factors released by necrotic cells are recognized and captured by phagocytes, triggering subsequent immune responses [34]. TNF-α released by activated neutrophils or other cells has also been shown to induce neutrophil necroptosis. In gout, neutrophil necroptosis drives the progression of the disease. Therefore, understanding how neutrophil necroptosis initiates inflammatory responses may provide therapeutic strategies to mitigate the progression of various diseases.

### 3.3. Neutrophil Pyroptosis and Gout

Pyroptosis is a regulated inflammatory form of cell death mediated by inflammatory caspases, including caspase-1 in the canonical pathway and caspase-4/5/11 in the non-canonical pathway [57,58]. It is initiated by inflammasome activation. In the canonical pathway, danger signals promote inflammasome assembly, leading to caspase-1 activation, maturation of IL-1β and IL-18, and cleavage of gasdermin D (GSDMD). The N-terminal fragment of GSDMD oligomerizes in the plasma membrane to form pores, resulting in ion flux, cell swelling, membrane rupture, and release of inflammatory intracellular contents [59,60,61]. In the non-canonical pathway, intracellular LPS directly activates caspase-4/5 in humans or caspase-11 in mice, which also cleaves GSDMD and can secondarily activate NLRP3 signaling [60,61].

The relationship between neutrophils and pyroptosis is more complex than in macrophages. Neutrophils can produce mature IL-1β after inflammasome activation, but they are relatively resistant to rapid caspase-1-mediated lytic pyroptosis, which may help preserve antimicrobial functions during acute inflammation [62]. This relative resistance does not mean that neutrophils cannot undergo pyroptotic death. Under specific inflammatory conditions, neutrophils can still undergo GSDMD-dependent membrane pore formation and pyroptotic death, particularly when inflammasome activation is strong or prolonged [63,64,65]. Therefore, the apparent discrepancy between neutrophil resistance to pyroptosis and pyroptosis-associated neutrophil death reflects differences in stimulus strength, pathway engagement, and the extent of GSDMD-mediated membrane damage.

In gout, MSU crystals can induce size-dependent pyroptosis in bone marrow-derived neutrophils and macrophages through NLRP3 inflammasome activation and subsequent caspase-1 cleavage. Inhibition of GSDMD oligomerization by dimethyl fumarate attenuates MSU crystal-induced pyroptosis and reduces acute inflammation in gout mouse models [66,67].

### 3.4. Neutrophil Autophagy in Gout

Autophagy is a conserved lysosome-dependent catabolic pathway activated by cellular stress, including nutrient deprivation, infection, hypoxia, and inflammatory stimulation [34,68,69]. Although autophagy generally supports cell survival by degrading damaged organelles and recycling intracellular components, excessive or dysregulated autophagy may also contribute to cell death under certain inflammatory conditions [68,69]. Therefore, in neutrophils, autophagy should not be regarded simply as an antiapoptotic mechanism but rather as a context-dependent process that regulates the balance between survival, effector activation, and death.

Inflammatory mediators can activate the autophagic machinery in neutrophils, linking autophagy to neutrophil stress adaptation and inflammatory function [70]. Autophagy has been implicated in neutrophil degranulation and NET formation, although these responses are not entirely dependent on autophagy alone [71]. In the context of gout flares, MSU crystals can stimulate autophagy-related signaling in neutrophils and promote NET formation [72]. Mechanistically, autophagy may facilitate the intracellular remodeling required for NET release, while superoxide generation cooperates with autophagy during NET-associated neutrophil death [73] (Figure 2C).

This dual role may be particularly relevant in gout. On the one hand, MSU-induced autophagy and NET formation may amplify local inflammation during the early phase of a flare. On the other hand, when NETs aggregate, they can contribute to inflammation resolution by limiting inflammatory mediators and sequestering MSU crystals. Moreover, autophagy can modulate TNF-α-induced neutrophil apoptosis [74], suggesting that autophagy may influence neutrophil lifespan and the balance between persistent inflammation and resolution during gout flares.

### 3.5. Neutrophil NETosis in Gout

Activated neutrophils create a web-like extracellular structure known as neutrophil extracellular traps (NETs), which consist mainly of decondensed chromatin decorated with granular proteins. These structures provide an antimicrobial mechanism that complements the conventional phagocytic functions of neutrophils. NET release can occur through lytic and non-lytic pathways. The lytic, death-associated form is commonly termed NETosis; therefore, NET formation should not be regarded as synonymous with neutrophil death [75,76,77,78].

NET release is influenced by various stimuli, including pathogens, cytokines such as IL-8 and TNF-α, ROS, LPS, and chemical agents [75]. PAD4-mediated histone citrullination contributes to some forms of NET release, but PAD4 is not universally required, particularly in response to MSU crystals [76,78]. Within NETs, decondensed chromatin forms the structural framework to which granular proteins, including neutrophil elastase and myeloperoxidase, are attached [77]. In gout, MSU crystals can induce NET release through multiple stimulus-dependent pathways, including lysosomal interactions, autophagy-associated signaling, and caspase-11/cofilin-associated signaling [7,17,18,19]. Whether this process is lytic or non-lytic may depend on the stimulus and cellular context.

## 4. NETs and Gout

### 4.1. NETs and Gout Flares

**Figure 3:**
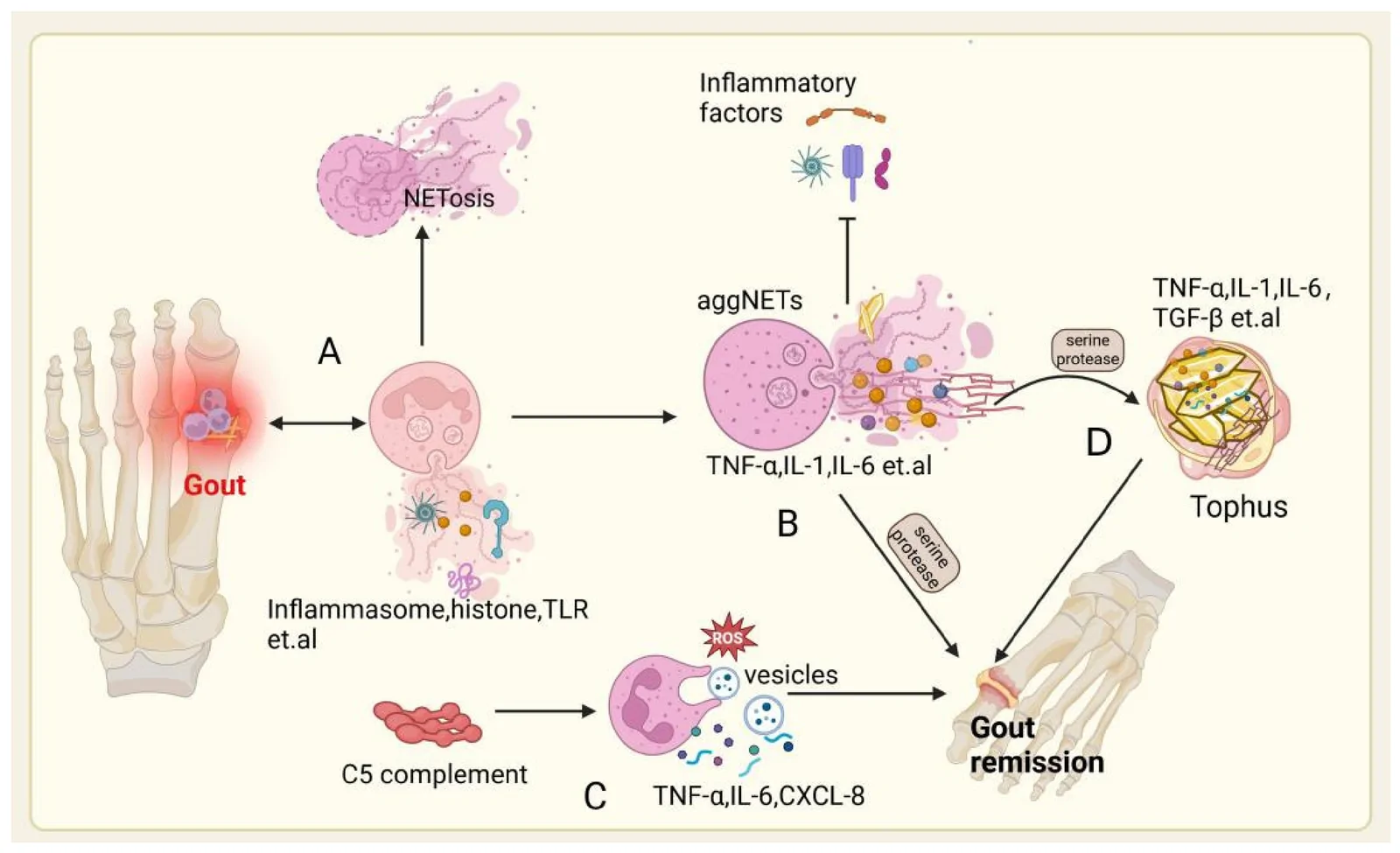
(**A**) NETs release histones, elastase, myeloperoxidase, and other granular proteins, which can amplify the local inflammatory response and may contribute to gout-associated pain [81]. Additionally, the formation of NETs is often accompanied by neutrophil death, a process defined as NETosis. (**B**) As the number of NETs increases, they aggregate, forming aggNETs. These aggNETs tightly bind to MSU crystals and counteract proinflammatory factors. AggNETs can quickly capture and cleave proinflammatory factors and absorb large amounts of inflammatory and chemotactic factors, such as TNF-α, IL-1, IL-6, and monocyte chemoattractant protein-1. Finally, serine protease degrades and inactivates these factors, leading to a “silent” state of gout. (**C**) During inflammation, neutrophils extend pseudopods to engulf MSU, forming phagosomes. This process is accompanied by a respiratory burst, producing large amounts of ROS and releasing inflammatory mediators such as TNF-α, IL-6, and the chemokine CXCL-8. At this time, neutrophils, under the influence of complement component 5, alleviate gout by releasing vesicles. (**D**) A tophus has a three-layer structure: the center consists of MSU crystals, the middle layer is immune cells, and the outer layer is connective tissue. Tophi typically contain anti-inflammatory and proinflammatory proteins, including IL-1β, IL-6, TNF-α, myeloid-related proteins 8 and 14, and TGF-β. The formation of tophi promotes the resolution of inflammation.

When MSU crystals deposit in the joints, they trigger a cascade of inflammatory responses. These deposited MSU crystals stimulate neutrophils to generate NETs, which have a dual impact on the development of gout [11]. This dual impact refers to the ability of NETs to amplify local inflammation during acute flares, while aggregated NETs can help limit inflammation by trapping MSU crystals and degrading proinflammatory mediators. Although PAD4-mediated histone citrullination is an important mechanism in some forms of NET formation, synovial fluid PAD activity is nearly absent in gout compared with RA and JIA [79,80]. A critical study has identified that NET formation requires the involvement of caspase-11. Neutrophils, which are heavily recruited to the joint cavity during a gout flare, are activated and induce NET formation in a caspase-11-dependent manner [19]. NETs release histones, elastase, myeloperoxidase, and other granular proteins, which can amplify the local inflammatory response. NET release may occur with or without neutrophil death; the lytic, death-associated form is commonly termed NETosis [81]. Furthermore, NETs induced by MSU crystals have been demonstrated to inhibit osteoblast activity while enhancing osteoclast activity [82] (Figure 3A).

Furthermore, MSU crystal-induced NETs have been shown to inhibit osteoblast activity while enhancing osteoclast activity [83]. This dual effect may contribute to bone erosion and joint damage in gout by disrupting bone homeostasis. The complexity of NETs is further underscored by their capacity to induce trained immunity, implying that NET-driven diseases may possess a memory component with crucial therapeutic implications [84]. These findings highlight the significant and complex role of NETs in gouty arthritis.

### 4.2. NETs and the Remission of Gout Flares

During the inflammatory response in gout, activated neutrophils release many proinflammatory mediators, attracting and activating more immune cells, thus forming a positive feedback loop. However, this process must be tightly regulated to avoid excessive inflammation. Specifically, regulatory T cells induced by B cells can reduce the influx of neutrophils and decrease NET formation, thereby alleviating inflammation [85]. Synovial macrophages also help resolve MSU crystal-induced inflammation by clearing NETs [84]. Neutrophil elastase associated with NETs can degrade proinflammatory cytokines, positively mitigating inflammation [86].

NETs are highly adhesive and effectively trap pathogens, enhancing their ability to kill bacteria. In gout, NETs similarly adhere to MSU crystals, akin to their trapping of bacteria. During the late stages of a gout flare, continuous recruitment of neutrophils to MSU deposition sites results in the release of proinflammatory cytokines such as TNF-α, IL-8, and IL-1β, which further stimulates NET release [87]. As NETs accumulate, they aggregate into structures called aggregated NETs (aggNETs). These aggNETs tightly bind to MSU crystals and increase serine protease levels, counteracting proinflammatory cytokines [11,88]. AggNETs efficiently capture and degrade large amounts of inflammatory and chemotactic factors like TNF-α, IL-1, IL-6, and monocyte chemoattractant protein-1 through serine proteases, thereby breaking the cycle of inflammation [44,89]. This mechanism contributes to inflammation resolution, leading gout into a “silent” state (Figure 3B). The appearance of aggNETs marks a pivotal moment in gout flares [90].

Additionally, several endogenous anti-inflammatory pathways may contribute to the resolution of gout flares. IL-37 has been reported to limit MSU crystal-induced innate immune responses [91], and IL-1 receptor antagonist (IL-1Ra) can counterbalance IL-1-mediated inflammatory signaling [92]. TGF-β1 production can inhibit neutrophil oxidative bursts and reduce NET formation [44]. Mechanistically, TGF-β signals through TβR-II/TβR-I and downstream Smad-dependent pathways to regulate inflammatory gene expression [93,94,95]. As an anti-inflammatory cytokine, IL-37 can suppress proinflammatory cytokine production [96]. In gouty inflammation, miR-223 can suppress IL-1β and TNF-α production by targeting NLRP3 inflammasome signaling [97]. Nrf2 antioxidant signaling has also been reported to alleviate gout arthritis pain and inflammation [98]. Together, these pathways may help restrain excessive NET-associated inflammation and promote the resolution of gout flares.

Oxidative stress is another crucial mechanism in gout pain and inflammation [99]. MSU crystals activate human neutrophils to produce ROS [100]. IL-33 promotes neutrophil influx and ROS production through the ST2 receptor [101]. NETosis induction depends on ROS production, primarily from NADPH oxidase [102]. Therefore, understanding the relationship between NETs and gout remission also requires considering the impact of oxidative stress and ROS.

### 4.3. Controversial Issues Surrounding NETs in Gout

Despite extensive research on the relationship between NETs and gout, several fundamental questions remain unresolved regarding NET formation mechanisms, including both NADPH oxidase-dependent and independent pathways. Although the critical role of aggNETs in resolving inflammation has been confirmed, the regulatory mechanisms behind their formation are still poorly understood. Key questions persist regarding whether NETs form primarily within tissues or in the bloodstream and what internal and external factors influence specific NET pathways [75].

In the context of MSU crystal stimulation, it has been observed that chronic granulomatous disease patients and NADPH oxidase-deficient mice exhibit reduced formation of aggNETs, highlighting the importance of ROS in MSU crystal-induced aggNET formation [88]. However, other studies have shown that MSU crystals can trigger NETosis through lysosomal interaction, independent of ROS and NADPH oxidase [8]. Additionally, while the regulatory role of the RIPK3/MLKL pathway in MSU crystal-induced NETosis is debated, inhibitors targeting this pathway do not significantly impact NET formation. Various factors such as adenosine triphosphate, lactoferrin, IL-1β, and the P2Y6 receptor antagonist MRS2578 may influence the formation of MSU crystal-induced aggNETs [88,103,104]. Further studies revealed that CD14+ macrophages in the synovial fluid of gout patients play a critical role in clearing MSU crystal-induced NETs. These ingested NETs can persist within macrophages without degradation [84]. Moreover, neutrophils deficient in MPO or NE can still respond to MSU crystals by releasing NETs, although they do not respond similarly to phorbol 12-myristate 13-acetate (PMA) [100,103]. It has also been demonstrated that the size of MSU crystals affects the formation of NETs and aggNETs, a process promoted by adding IL-1β [62]. Notably, the composition and DNA origin of MSU-induced NETs remain partially understood and appear to differ from NETs induced by other stimuli [7]. These findings offer new perspectives on how MSU crystals induce NETs, underscoring the complexity and variability of NET formation mechanisms in gout. 

## 5. Neutrophil-Derived Extracellular Vesicles

Neutrophils also alleviate gout by releasing extracellular vesicles (EVs) [105,106], a process triggered by macrophage-derived complement component 5 [107]. As the most abundant polymorphonuclear leukocytes in the synovial fluid of gout patients and the primary effector cells of the innate immune system, neutrophils are attracted to inflammation sites by chemotactic factors. There, they extend pseudopods to engulf MSU crystals, forming phagosomes. This process involves an oxidative burst, producing large amounts of ROS and releasing inflammatory mediators such as TNF-α, IL-6, and the chemokine CXCL-8. Exosomes have been implicated in the pathological processes of gouty arthritis, including MSU crystal-associated inflammation, immune cell activation, inflammasome regulation, and inflammatory resolution [108] (Figure 3C). Research indicates that EVs on the surface of neutrophils, stimulated by MSU in vitro and a peritonitis mouse model, can enter cartilage and protect joints. These EVs, rich in phosphatidylserine, can inhibit NLRP3 inflammasome-related IL-1β production and affect the responses of macrophages and dendritic cells to zymosan A and LPS, promoting the release of the anti-inflammatory cytokine TGF-β1 from macrophages [109].

In vitro experiments have also shown that neutrophils, under various stimuli such as inflammatory mediators (TNF, IFN-γ, GM-CSF, and IL-8) and bacterial molecules (e.g., Mycobacterium tuberculosis, Neisseria meningitides, fMLP, LPS), increase their exosome release [110,111]. However, studies have also demonstrated that MSU-stimulated neutrophil-derived exosomes can increase the expression of miR-1246. Overexpression of miR-1246 reduces alkaline phosphatase and osteoprotegerin levels while increasing the expression of RANKL. This MSU-stimulated neutrophil-derived exosome-mediated increase in miR-1246 expression inhibits osteoblast activity [112]. Nonetheless, the specific anti-inflammatory mechanisms of MSU-induced exosomes still require further investigation.

## 6. Neutrophils and Tophus Formation

Tophi are granuloma-like structures formed by the encapsulation of MSU crystals by mononuclear and multinucleated giant cells. The core is composed of MSU crystals, which induce a chronic foreign body reaction and are surrounded by epithelial cells and macrophages, forming a foreign body nodule. Recent studies have found that neutrophil NETs are a significant component of tophi. Remarkably, embedding MSU crystals in aggNETs may underlie tophus deposition, potentially impacting the spontaneous remission of gout [89]. When the body fails to eliminate MSU crystals, these crystals are encapsulated by monocytes and multinucleated giant cells, forming tophi [113]. Tophi are a hallmark of chronic gout and have a complex formation mechanism. They consist of three layers: a central core of MSU crystals, an intermediate layer of immune cells, and an outer layer of connective tissue [114]. Anti-inflammatory and proinflammatory proteins, including IL-1β, IL-6, TNF-α, myeloid-related proteins 8 and 14, and TGF-β, are typically present in tophi [115]. These inflammatory mediators and proteins indicate an inflammatory response occurring with MSU crystal deposition. Subsequently, NETs aggregate into aggNETs, which degrade inflammatory factors via serine proteases and generate anti-inflammatory proteins to mitigate inflammation [116] (Figure 3D).

During the formation of tophi, aggNETs play a crucial role by encapsulating MSU crystals in granuloma-like structures, leading to an “immunosilent” state. However, changes in environmental factors such as blood uric acid levels, temperature, and hydrogen potential may disrupt this balance. Over time, the interaction between aggNETs and MSU crystals forms a complex multilayer structure, eventually developing into visible tophi. Under normal circumstances, the body produces Deoxyribonuclease I (DNase I), an enzyme that degrades NETs, maintaining their dynamic balance. NETs can upregulate NLRP3 and pro-IL-1β levels, induce ROS production, and activate the NLRP3 inflammasome. The degradation of NETs by DNase I can mitigate this activation process [117].

Interestingly, research by Chatfield et al. [8] showed that unlike PMA and Candida, crystals such as MSU can induce the formation of NETs resistant to DNase I degradation, making the MSU crystals encapsulated by aggNETs harder to clear. Over time, the combination of NET polymers and MSU crystals forms complex structures, ultimately leading to the formation of tophi, which indicates that aggNETs play a critical role in the formation of tophi and profoundly impact disease progression, extending beyond the remission of acute inflammation.

## 7. Conclusions and Perspectives

Neutrophils play a complex and critical role in the development of gout. Although gout involves imbalances in various blood cell proportions [25], gout is primarily characterized by massive neutrophil infiltration [20,21,22]. Neutrophils not only trigger and guide the inflammatory process in gout but also exhibit anti-inflammatory effects during the acute phase through aggNETs. The interaction between MSU crystals and cells within the joint promotes the recruitment and activation of neutrophils, which release inflammatory mediators, induce the formation of NETs, and release DAMPs. During the resolution phase of inflammation, macrophages clear apoptotic neutrophils, and the interaction between these apoptotic neutrophils promotes the production of the anti-inflammatory factor TGF-β. MSU crystals may also lead to various pathological changes in neutrophils, including excessive autophagy, necroptosis, pyroptosis, and efferocytosis. However, considering the existence of asymptomatic hyperuricemia, our understanding of the role of the immune response to MSU remains incomplete.

Treatment strategies targeting neutrophils and NETs have yet to progress significantly. Nevertheless, neutrophils and NETs are still considered potential therapeutic targets for gout due to their inherent antimicrobial and immunoregulatory properties. Further exploration of the potential roles of NETs remains a crucial direction for future research. Recent studies have provided new insights. For example, treatment strategies based on antagomir-3146 have effectively inhibited the formation of NETs, revealing the potential regulatory role of miR-3146 in the pathogenesis of gout [118]. Another study explored the inhibitory effect of anakinra on NET formation, which showed dose- and time-dependent effects by regulating intracellular calcium mobilization in PMA-stimulated neutrophils in vitro [119].

Additionally, research by LIU et al. [120] found that the mRNA levels of the GPR105 gene (a P2Y14 receptor closely related to neutrophil activation and inflammation) were significantly higher in neutrophils from patients with acute gout attacks than in those with asymptomatic hyperuricemia and healthy volunteers. Targeting GPR105 can promote the transition between neutrophil NETosis and apoptosis, thereby reducing inflammation and joint damage caused by gout. JATI et al.’s research [121] revealed an anti-inflammatory phytochemical inhibitor: piperine. This substance can alleviate inflammatory responses by regulating the role of the NLRP3 inflammasome in gouty inflammation, inhibiting tophus formation caused by NETosis, and reducing cartilage erosion and leukocyte infiltration.

However, it is essential to note that neutrophils play a crucial role in host defense. Therefore, any therapy targeting NET formation must avoid impairing the physiological functions of these cells. We could consider targeting neutrophil function and death processes in future gout prevention and treatment research. Strategies such as inhibiting the excessive activation of neutrophils and preventing their accumulation in joints [122], promoting the formation of large amounts of aggNETs, or facilitating the clearance of apoptotic neutrophils could provide new ideas and methods for gout prevention and treatment.

Alongside these experimental strategies, current gout management follows a treat-to-target strategy aimed at reducing the urate burden and controlling acute inflammation. Allopurinol is recommended as the preferred first-line urate-lowering therapy, while febuxostat, uricosuric agents, and pegloticase may be used in selected patients according to disease severity, comorbidities, and treatment response. Colchicine, nonsteroidal anti-inflammatory drugs, and glucocorticoids remain the main treatments for acute gout flares. IL-1-targeted agents, including anakinra and canakinumab, may be considered in selected patients when conventional anti-inflammatory treatments are contraindicated, poorly tolerated, or ineffective [123,124]. These therapies may indirectly reduce neutrophil recruitment and activation by lowering the MSU crystal burden or suppressing IL-1-dependent inflammatory signaling; however, they do not specifically target neutrophil death programs or NETosis.

Translational development is also moving toward innate immune targets. Dapansutrile (OLT1177), an oral NLRP3 inhibitor, is currently being evaluated in a multicenter phase 2/3 randomized, placebo-controlled clinical trial for acute gout flares (NCT05658575) [125]. The trial is designed to assess the efficacy and safety of dapansutrile in controlling acute gout symptoms and pain, rather than NETosis or neutrophil fate as primary endpoints. Therefore, direct clinical evidence for therapies targeting NETosis, aggregated NETs, or neutrophil death programs remains limited. Future clinical studies should determine whether selective modulation of neutrophil recruitment, NET release, NET clearance, or the transition between NETosis and apoptosis can improve gout outcomes without compromising host defense.

## Data Availability

No new data were created or analyzed in this study. Data sharing is not applicable to this article.

## Abbreviations

The following abbreviations are used in this manuscript:

MSU	Monosodium Urate
NETs	Neutrophil Extracellular Traps
IL	Interleukin
NLRP3	Nucleotide-Binding Oligomerization Domain-Like Receptor Protein 3
NF-kB	Nuclear Factor Kappa-Light-Chain-Enhancer of Activated B Cells
TLR2	Toll-like Receptor 2
TLR4	Toll-like Receptor 4
aggNETs	Aggregated NETs
TNF	Tumor Necrosis Factor
DNA	Deoxyribonucleic Acid
PI3K	Phosphoinositide 3-Kinase
IFN-γ	Interferon-γ
TGF	Transforming Growth Factor
GM-CSF	Granulocyte–Macrophage Colony-Stimulating Factor
RIPK3	Receptor-Interacting Protein Kinase 3
MLKL	Mixed Lineage Kinase Domain-Like Protein
ROS	Reactive Oxygen Species
GSDMD	Gasdermin D
LPS	Lipopolysaccharide
BMNs	Bone Marrow-Derived Neutrophils
MPO	Myeloperoxidase
PAD	Peptidyl Arginine Deiminase
PMA	Phorbol 12-Myristate 13-Acetate
C5	Complement Component 5
EVs	Extracellular Vesicles
